# Exploring Older Adults’ Needs for a Healthy Life and eHealth: Qualitative Interview Study

**DOI:** 10.2196/50329

**Published:** 2025-01-08

**Authors:** Paula Valkonen, Sari Kujala, Kaisa Savolainen, Riina-Riitta Helminen

**Affiliations:** 1 Department of Computer Science Aalto University Espoo Finland; 2 Suomen Terveystalo Oy Suomen Terveystalo Oy Helsinki Finland; 3 Department of Industrial Engineering and Management Aalto University Espoo Finland

**Keywords:** older adults, eHealth, needs, retirement, well-being, cultural probes, sentence completion, human-computer interaction

## Abstract

**Background:**

Aging brings physical and life changes that could benefit from eHealth services. eHealth holistically combines technology, tasks, individuals, and contexts, and all these intertwined elements should be considered in eHealth development. As users’ needs change with life situations, including aging and retirement, it is important to identify these needs at different life stages to develop eHealth services for well-being and active, healthy lives.

**Objective:**

This study aimed to (1) understand older adults’ everyday lives in terms of well-being and health, (2) investigate older adults’ needs for eHealth services, and (3) create design recommendations based on the findings.

**Methods:**

A total of 20 older adults from 2 age groups (55 to 74 years: n=12, 60%; >75 years: n=8, 40%) participated in this qualitative interview study. The data were collected remotely using a cultural probes package that included diary-based tasks, sentence completion tasks, and 4 background questionnaires; we also performed remote, semistructured interviews. The data were gathered between the fall of 2020 and the spring of 2021 in Finland as a part of the Toward a Socially Inclusive Digital Society: Transforming Service Culture (DigiIN) project (2019 to 2025).

**Results:**

In the daily lives of older adults, home-based activities, such as exercising (72/622, 11.6% of mentions), sleeping (51/622, 8.2% of mentions), and dining and cooking (96/622, 15.4% of mentions), promoted well-being and health. When discussing their needs for eHealth services, participants highlighted a preference for a chat function. However, they frequently mentioned barriers and concerns such as the lack of human contact, inefficiency, and difficulties using eHealth systems. Older adults value flexibility; testing possibilities (eg, trial versions); support for digital services; and relevant, empathetically offered content with eHealth services on short-term and long-term bases in their changing life situations.

**Conclusions:**

Many older adults value healthy routines and time spent at home. The diversity of older adults’ needs should be considered by making it possible for them to manage their health safely and flexibly on different devices and channels. eHealth services should adapt to older adults’ life changes through motivation, personalized content, and appropriate functions. Importantly, older adults should still have the option to not use eHealth services.

## Introduction

### Background

Aging involves physical and life changes [1-4], where eHealth services have the potential to provide support and benefits [4-7]. The prevalence of many diseases increases with age [8,9], and multimorbidity is common among people aged ≥65 years [10,11]. Conversely, healthy behaviors can extend people’s lives [12-14].

eHealth can help people nurture their health and well-being, potentially extending their lives. eHealth can also make treatment more accessible, promote treatment continuity, enhance communication, help shared decision-making, and enable patient self-management [10]. Older adults seem to be generally positive toward eHealth in a health management context [15]. They can benefit from eHealth services, as they often have complex health issues and engage in many self-management tasks supporting their health and well-being [1,5,16].

However, older adults sometimes face challenges with eHealth services [5,16,17], including a lack of skills and interest [3,16,18]. Hirvonen et al [19] reported that some older adults find the content or functionalities of eHealth services irrelevant (eg, [20,21]). Sometimes, older adults might even feel that digital services are not “meant for them” (eg, [20,22]). Thus, older adults might have a risk of exclusion from digital services [16,18,23,24] and society [16,23].

Older adults have not always been involved in technological development, especially in mainstream technology [25-27]. At the same time, the older population is growing [28], and health care costs are rising [29]. Stephanidis et al [30] encourage understanding emerging new technology possibilities and context-based user needs when designing digital solutions for the health and well-being (hereafter, *eHealth services* or *eHealth*) of older adults and a better understanding of the role of technology in older adults’ lives [30]. Ideally, the technology should be harmonized with these adults’ lives [15].

European older adults regularly engage in work, household chores, exercise, cultural activities, and tourism [31]. On the basis of the literature, many older adults in Europe live in rural areas, where they have easy access to nature and can engage in refreshing activities [31]. On the other hand, older adults with urban lifestyles access a variety of services [31]. For example, in Finland and Sweden, rural areas are in the north and distant from health care services, and eHealth services offer a promising option for taking care of one’s health [32]. Physical activities might support older adults’ health situation during aging. In 2017, in total 43.2% of Europeans aged 50 to 64 years and 44.5% of those aged 65 to 74 years old spent >3 hours per week engaging in physical activity [31]. On the other hand, older adults’ daily routines differ. Older adults are a heterogeneous group of people, and aging is a process, not a static state [33]. Therefore, it is important to obtain qualitative insights into older adults’ activities. These could help inform the idea and role of eHealth services and harmonize it with older adults’ routines [15,30]. We know that older adults use eHealth services, for example, depending on their digital devices, capabilities, interests, or education [31,34]. Statistically, 100% of Europeans have, at least in theory, access to eHealth services or health information [35].

The best approach to eHealth service development means selecting the optimal approach case by case [36]. In eHealth, technology, tasks, individuals, and contexts are holistically combined, which should all be considered in developments [36-39]. eHealth services should genuinely support people pursuing healthier lifestyles and be accessible to all [10], although older adults may perceive the technology differently from other generations [1,40]. Humanity is needed alongside IT. Therefore, design decisions should also help ensure that as a part of eHealth services development, the human touch of personal care will not be removed [30,41].

Mapping user needs is important because without understanding needs, designing high-quality solutions is challenging [42]. User needs can be defined as the problems that prevent users from reaching their goals or possibilities to support them in reaching these goals [43]. User needs can be personal or more social in nature, and they can be related, for example, to available information or technology functions [44]. Poor understanding of users, user needs, and use contexts increases the risk of failure in eHealth service development [39]. On the other hand, user needs are not a stable phenomenon; they might change with age or the use of technology [45]. However, not much is known about what kind of user needs for eHealth services arise in older adults’ lives, which is unfortunate because understanding user needs is a great innovation source [46]. In addition, Hirvonen et al [19] recommend further investigation into older adults’ eHealth service use as a part of their daily lives.

### Goal of This Study

We aimed to understand older adults’ everyday lives in terms of well-being and health and their needs for eHealth services. We investigated 2 life stages: working at an older age and life after retirement, possibly with a chronic disease. We were interested in the health and well-being practices of 2 age groups to understand older adults’ needs for eHealth services. Investigating these aspects allows developers and designers to better understand how eHealth services can meet older adults’ needs and support their healthy living.

Users’ needs change with life situations, including aging [47]. Therefore, if we want to harmonize eHealth services with older adults’ everyday use, as is recommended by Cabrita et al [15], and to support technology development for well-being and an active, healthy lifestyle, it is important to identify user needs at different stages of life and daily routines in different life situations. The research questions were as follows:

1. What is everyday life like for older adults?
2. What kind of needs do older adults have for eHealth services?

### Prior Work

#### Qualitative Methods for Investigation of Needs

User needs can be explored in many ways. In the human-computer interaction field [48,49], designers and researchers have several methods for investigating user needs. Soraghan et al [50] and Dickinson et al [51] encourage researchers and designers to step into older adults’ homes to assess technology use and empathize with respondents’ lives, for example, through in-home interviews and observations. Understanding of older adults can be collected through remote, semistructured interviews [52]. Projective techniques, such as cultural probes [53-57] or sentence completion tasks [58], in which the participants provide information about themselves (instead of the researcher asking them direct questions), can be fruitful methods for gaining a deeper understanding of end users when the topic is challenging to verbalize or includes sensitive aspects [42]. For example, the sentence completion technique can be used to gather both an understanding of end users’ values and needs as well as inspirational data for design and discussion activation [42,59,60].

Cultural probes are designed to trigger older adults to provide inspirational information about their lives to designers [53]. Following development, they have been widely used for various purposes, including collecting information about caregivers [56], investigating patient experiences with an eHealth service [54], and in other health care contexts [55,57]. Cultural probes are packages or toolkits of various tasks that end users complete and document [53]. While cultural probes show potential as a projective technique for gathering user needs [42], they appear to be underused in identifying older adults’ needs for eHealth services.

#### Older Adults’ Needs and Attitudes Toward Technology

Older adults’ needs and attitudes toward technology vary. For example, some older adults do not trust computers and find the terminology and content of computers and digital services confusing [17,18,61]. They might even fear computers [61]. Their self-confidence with computers is sometimes fragile, and usability issues can be disastrous for their self-confidence [61]. In addition, older adults as a user group are much more diverse than many traditional user groups; they may have problems such as sensory loss, challenges with language, and attitudes to technology [61].

Many older adults appreciate easy access to eHealth services and their medical records, which should be offered in an easy, understandable, and secure way [5,52]. When medical information has compact and clearly labeled contents in well-organized menus [62], clear interface language, and linear navigation [20], this can assist older adults. Backonja et al [47] recommend eHealth services that adjust functionality and content based on the user’s needs. Similarly, Cabrita et al [15] recommend personalization of design and functionality, thus lowering older end users’ fears toward technology by allowing it to support its users and even offer empathy and sympathy to them.

## Methods

### Overview

**Figure 1:**
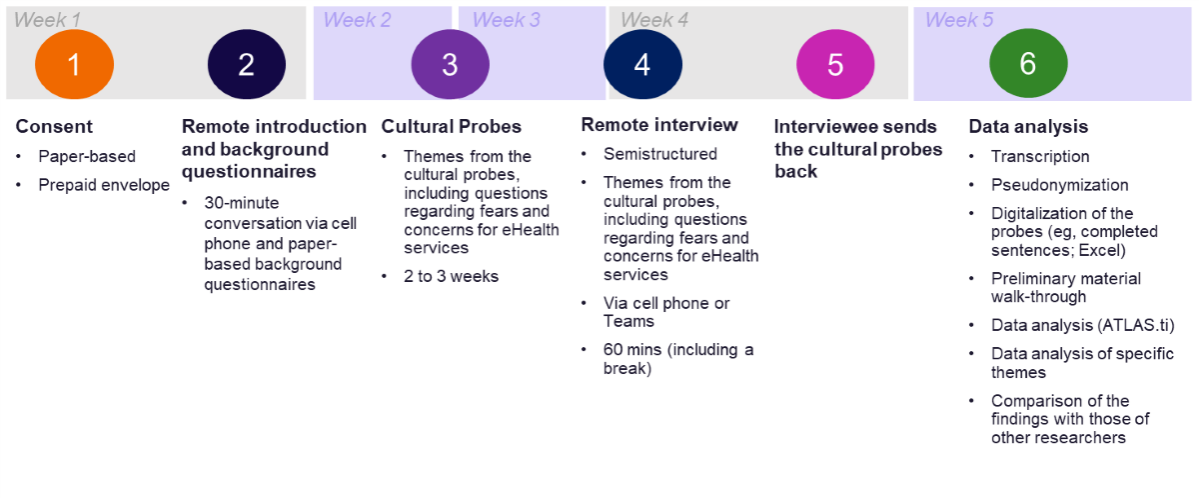
Research procedure.

This empirical qualitative study consisted of cultural probes [53,63], which included 4 background information questionnaires (*background questionnaires*), sentence completion tasks [42,58,59], and a diary-based exercise [64-66], and remote interviews [52]. Data collection included 6 phases, which all played an important role (Figure 1). Data collection started with filling out a consent form. After that, background information was collected, and a short remote interview was conducted. Then, the participant had time to respond to the cultural probe package (ie, sentence completion tasks and diary-based exercise). Participation ended with a long remote interview and the return of the probes package to the researcher. Finally, the data were analyzed.

### Sampling and Recruitment

The research was conducted in Finland between the fall of 2020 and the spring of 2021 (ie, during the COVID-19 pandemic) as part of the DigiIN project (2019 to 2025).

Defining an older adult purely by age is somewhat complex [67]. Generally, they are people in late adulthood. In line with the study by Ware et al [5], this study focused on older age groups associated with a higher risk of chronic disease, with participants from 2 age groups (those aged between 55 and 74 years and employed and those aged >75 years and retired). Group 1 participants (ie, those who were still working) were relevant because aging includes preparing for retirement [68].

The participants were selected through purposive sampling (digital survey) and snowball sampling [69]. Group 1 participants (12/20, 60%) were recruited via a digital survey [70]. Group 2 participants (8/20, 40%) were recruited in 2 ways: via the digital survey and snowball sampling. To ensure a diverse and engaged sample for this long-term study, participants’ interest in digital services was assessed in advance. The groups were formed according to the life situation: group 1 participants were still working, and group 2 participants were retired. The age category of group 1 was between 55 and 74 years (later, 55-74 years). The age category of group 2 was between 75 and >90 years (later, >75 years). Participants in group 1 had different professions, such as yoga teacher, director, project manager, or entrepreneur. The participants were from different parts of Finland but did not fully cover the whole country.

After participants expressed potential interest in participating in the study, the researcher explained the study in a telephone call. This call lasted for 15 to 30 minutes, depending on the participant’s questions. If the participant was still interested, the researcher mailed or emailed the information letter that described this study in detail and a consent form to the participants for their signature.

### Ethical Considerations

The empirical study was conducted as part of the DigiIN project (2019 to 2025). The study protocol was reviewed and approved by the Ethical Review Board of Aalto University (95_03.04_2019_DigiIN). All participants provided their voluntary, informed, and written consent. Patients’ ability and willingness to participate in the study were confirmed through a phone conversation before they signed the consent form. During the call, the study process was explained, and it was emphasized that participation was voluntary, with the option to withdraw at any time. This was also mentioned in the written consent form.

### Research Procedure

The study comprised six phases (Figure 1):

1. First, the study purpose and research procedure were introduced.
2. Second, the cultural probe packages, including 4 background questionnaires, were delivered. The study procedure and contents of the package were explained again in detail by phone or Teams (version 1.6.00.11166; Microsoft Corporation).
3. Third, the participants filled out the cultural probes for 2 weeks, during which the researcher made 2 short phone calls to the participants, being present for possible questions and collecting specific day-based information about the participants’ routines and activities related to health and well-being.
4. At the end of the study, the participants were interviewed remotely.
5. Then, they returned the probes package to the researcher in a prepaid envelope. The data collection took a maximum of 5 weeks. When the researcher received the probes package, the participants were sent a small thank-you gift.
6. Data were analyzed.

### Cultural Probes

#### Overview

In this study, cultural probes made data collection of older adults’ everyday lives possible in research environments, where remote data collection was important for safety reasons [52]. It helped in collecting data indirectly, as the participants themselves completed the cultural probes at their own pace as part of their everyday lives [42,52,53]. It also helped the participants prepare for the interview [52].

The cultural probe package, recruitment questionnaire, and recruitment letter were tested with 2 nonparticipating members of the target group. The probes, questionnaires, and letter were revised based on the feedback. The research materials were then tested again with another target group member. Finally, the entire study setup with semistructured interviews was tested with 2 target group members.

#### Background Questionnaires

Among other tasks, the cultural probe package included 4 questionnaires: a background information questionnaire, the Health Confidence Score (HCS) [71], the eHealth Literacy Scale (eHEALS) [72], and the European Health Literacy Survey Questionnaire (HLS-EU-Q16) translated into Finnish by Eronen et al [73]. The other questionnaires were translated into Finnish by the authors. Questions 3 to 10 in the eHEALS questionnaire were used in this study with the added option of “I don’t know” [72].

In addition to demographic questions, the background information questionnaire asked about the participant’s internet and device use, the eHealth services they had used, and how useful they found them. The purpose of the HCS, eHEALS, and HLS-EU-Q16 questionnaires was to obtain an overview of the participants’ capabilities (or perceived skills) to understand health information and use eHealth services.

#### Diary-Based and Sentence Completion Tasks

The cultural probe package (1) motivated participants to notice and document their actions related to health and well-being as a part of their everyday lives and (2) worked as a triggering element for remote, semistructured interviews. In this remotely conducted study, cultural probes and their exercises helped make the researcher appear more relatable to the participant. This was especially important given the sensitive and personal topics, such as everyday life around health and well-being, discussed in the study, both from the researcher’s perspective, and more importantly, from the participant’s perspective [74]. The cultural probe tasks included sentence completion tasks [59] and a diary-based task [64-66]. In the diary-based task, participants were asked to make notes regarding their health routines, decisions, and actions for 5 days. In addition, 13 incomplete sentences were formulated following the best practices explained by Nurkka et al [42] and included in the pretesting phase. In the sentence completion task, participants were asked to complete sentences (Multimedia Appendices 1 and 2) with 4 themes (Table 1).

**Table 1:** Themes of the sentence completion tasks.

Theme	Included in group 1	Included in group 2
eHealth services now	Yes	Yes
eHealth services in the future	Yes	Yes
Concerns and fears for eHealth services	No	Yes
Informal caregiver’s eHealth services (excluded from the data)	Yes	Yes

The cultural probe packages had differences regarding incomplete sentence themes between the group 1 and group 2 participants. The study started with the younger participant group. As data began to accumulate, additional questions addressing concerns and fears were incorporated into the sentence completion tasks, as shown in Table 1. This could support the participants in preparing for the interview as well [52]. However, the interview covered the theme with both participant groups.

### Remote, Semistructured Interviews

The themes of the cultural probes were investigated in more detail in remote, semistructured interviews [52], which took 1.5 hours maximum to complete with group 1 participants and 1 hour maximum with group 2 participants. If an interview took longer, the participants were offered the opportunity to continue the interview the next day. The researcher made phone calls using either the Teams call function or a mobile phone, making it easier for participants to take part. Two researchers conducted the interviews, but only 1 conducted each interview for trust-building purposes. The interviews were audio recorded and documented with field notes.

The interviews comprised different perspectives regarding the participants’ everyday lives and activities concerning health and well-being. The interview structure (Multimedia Appendix 3) was created based on the themes of the cultural probes. The participants were asked to describe the contents of their cultural probes. The idea was to empower the participants and let them decide how much or in how much detail they were willing to express their thoughts on their well-being and everyday lives. In the interview design, the interview checklist by Tong et al [75] was followed.

### Analysis

#### Overview

After the data were transcribed and pseudonymized, the background questionnaire was analyzed using descriptive statistics, and the qualitative data were analyzed following the inductive content analysis process [69,76,77]. User needs were formulated based on the content analysis of the interviews and the analysis of the cultural probe data. The cultural probe data included the participants’ diary notes of their daily well-being activities and completed sentences. The data were analyzed according to data type. The different analysis phases are explained in the subsequent sections.

#### Preliminary Material Walk-Through

During the interviews, a researcher (PV) identified the preliminary content-based categories and, after each interview, categorized each participant’s direct quotes in Excel (version 2208 Build 16.0.15601.20644; Microsoft Corporation). Due to the richness of the data, an additional preliminary review was necessary. The researcher (PV) manually reviewed the entire dataset through after receiving the cultural probes package, using traditional pen-and-paper data analysis methods [78].

After this preliminary data analysis phase, the analysis needs for the next phases were recognized. The analysis needs were identified, keeping in mind that the purpose was to understand user needs. Similar to the data analysis process proposed by Nielsen et al [79], the preliminary material walk-through process needed both systematic and circular practices.

#### Content Analysis for Interview Data

Afterward, 2 researchers (PV and KS) read the pseudonymized interview transcripts and coded the data in ATLAS.ti 9 (version 9.1.5.0; Scientific Software Development GmbH; Multimedia Appendix 4) to understand the data as a whole and to support the analysis of the cultural probes data later. The process proposed by Mayring [76] was followed in the analysis. When approximately 30% (11/40) of the transcripts were coded, the researchers (PV and KS) revised and compared their categories and agreed on common ones. Finally, the results were discussed again by 2 researchers (PV and KS), and the findings were reported.

#### Analysis for Background Information Questionnaire Data

The background information questionnaires were analyzed to understand the participants’ characteristics and their internet and IT use habits. These questionnaires provided preliminary information about the sample. The questionnaire analysis was done in collaboration between 2 researchers (PV and SK). The means were calculated for the data from each of the 3 questionnaires (PV), and the results were discussed (PV and SK). Owing to the qualitative nature of the study and the small sample size, detailed quantitative analysis was not performed.

#### Content Analysis for Cultural Probe Data

The data from the cultural probe package were analyzed in collaboration between 3 researchers. Each sentence’s main content was analyzed by a researcher (PV), and the results were discussed with 2 other researchers (KS and SK) to ensure the quality of the analysis. The diary analysis focused on the participants’ descriptions of their well-being activities. Activities were written on sticky notes, 1 per note, with the participant code and time of day. After 2 researchers (PV and KS) created the notes for 1 participant, their consistency was checked: a researcher (KS or PV) read the findings and another one (KS or PV) wrote the findings down. The analysis included 644 grouped sticky notes. The sticky notes were grouped by 2 researchers. The notes were taken during the analysis process.

## Results

### Overview

The results are discussed in three parts: (1) participant information; (2) description of older adults’ everyday lives; and (3) user needs, which were expressed based on the findings from the sentence completion and remote, semistructured interview data.

The data comprised 19 cultural probe packages and 329 pages of transcripts of 20 interviews (in Verdana font, 11-point font size, single spaced). One participant did not return the cultural probe package.

### Participants

The study included 20 participants (Table 2). The participants were divided into two groups: group 1, those who were still employed, and group 2, those who were retired. Both groups included active computer users with many different devices, but compared to group 1, group 2 included more participants who did not routinely use the internet and IT. However, the variation in device types was greater in group 2 compared to group 1. The results suggest that perhaps retired people use health and well-being services less frequently in general than those who are working.

**Table 2:** Participant background information (N=20).

				Group 1 (n=12)		Group 2 (n=8)
Age (y)				55 to 74		>75
**Gender, n (%)**						
	Women		9 (75)		6 (75)	
	Men		3 (25)		2 (25)	
**Internet activity, n (%)**						
	Internet use daily or many times in a day		11 (92)		6 (75)	
	Internet use on a weekly basis		1 (8)		1 (12)	
	Do not know to use or do not use the internet		0 (0)		1 (12)	
**Internet habits, n (%)**						
	**Most popular devices for internet use**					
		Computer	12 (100)		5 (63)	
		Cell phone	12 (100)		4 (50)	
		Tablet	9 (75)		4 (50)	
	**Place of internet use**					
		At home	12 (100)		7 (88)	
		Outside home	10 (83)		3 (38)	
	**The most popular reasons to use the internet**					
		Searching information	12 (100)		7 (88)	
		Banking	12 (100)		6 (75)	
		Health and well-being services	12 (100)		4 (50)	
**eHealth use, n (%)**						
	**Most popular digital public health care services used**					
		Patient portal: checking health information and receipt renewal	12 (100)		4 (50)	
		Appointment booking	11 (92)		4 (50)	
		Chat, email, or SMS text message to health care professionals	8 (67)		—^a^	
		Visiting a health center’s web pages	—		2 (25)	

^a^Data not available.

All group 1 participants (12/12, 100%) and half (4/8, 50%) of the group 2 participants checked their health information and renewed their prescriptions via eHealth services. Internet users used it for banking and information searches, and 80% (16/20) of them also used it for health services.

Multimedia Appendix 5 presents the background questionnaire results. The eHEALS results (20/20, 100%) show the participants’ familiarity with using digitally offered health information, with differences between the participant groups. The results of the HLS-EU-Q16 (19/20, 95%) on health literacy indicate that the participants seemed to have at least basic health literacy skills. One participant did not send the questionnaire back to the researchers. The HCS results (20/20, 100%) on patients’ confidence in taking care of their own health show that the participants had at least a basic understanding of their health situation and how to operate in case of health challenges [63].

### Everyday Life of Older Adults

The analysis of the data from the diary-based task and the interviews resulted in 15 themes in the participants’ everyday activities. The themes were spending time at home, dining and cooking, routines, hobbies or exercising, sleep, mundane activities, work-related activities, medical treatment, cottage or nature, friends, pets, pampering and rest, communication with a relative, planning and controlling everyday life, and weather.

The most common activity was spending time at home, often watching television (102/622, 16.4%); spending time at home and dining and cooking (96/622, 15.4%) were the most popular regular activities of the participants. The importance of food for well-being was often mentioned in the data. Exercising was also popular (72/622, 11.6%), with walking and jogging being commonly practiced, including Nordic walking and climbing stairs. Walking could also be a social activity with a partner or a dedicated group. Sleep was mentioned in the data 51 out of 622 times (8.2%), as were mundane activities, such as going to the post office or having lunch or a coffee (51/622, 8.2%). The repetitive nature of routines, including spending time at home, the importance of food, and the variety of exercises, is reflected in the following quotes (the quotes are translated from Finnish):

From Monday to Friday, I wake up 5.50 am on two mornings, and on other mornings 6.50 am. And that 5.50 am means, that I go swimming. And on other mornings I eat my breakfast normally at home and go to work. I drink my morning coffee at the office.

Group 1, participant U2

There have been such beautiful days lately. Today it seems it is not so beautiful, or at least the sun is not shining yet. I feel I’m privileged that I’m able to live in my hometown, because this is such a beautiful city. I started with 30 min walking exercises, and now I do my walking exercise two times in week. Each walking exercise takes one and half hours nowadays. I explore those different terrains, and we have here a lot of a lot of stairs and a lot of small hills, which I use to improve my fitness.

Group 1, participant U12

I start my work at 9 am, then I eat breakfast, and walk with my dogs. I normally work at my computer until 6–8 pm. I eat, and drink coffee during short work breaks. At 4 pm I give food to my dogs. In the evening, I don’t meet anybody else than other dog owners. I also meet new employees during my workday.

Group 1, participant U6

I eat breakfast in the morning, and at the same time, I read news from my iPad. My husband and I have not ordered any paper newspapers.

Group 2, participant HX4

I might visit quite often to meet my neighbor, but not on an everyday basis. But it is a part of my everyday life. ...And then, I go quite often after 6 pm a short walk, and at the same time, visit at my friend, who has two cats. ...Then I make some dinner and watch TV News. I do not usually follow any TV series, but I might sometimes watch something from TV. I go to sleep at around 12 am.

Group 2, participant HX5

I don’t have so many meetings anymore, but my week starts with English lessons every Monday at 10 am. At 4 pm I go to an hour’s outdoor exercise. In the evening, I often go to German language speaking exercise group. Next day, I go to aqua jogging, and after that we take a cup of coffee with the members of that group. Next day I participate in outdoor walking exercise group, with which we normally walk an hour together. And at the end of the week, I often do some housework, a little bit cleaning home or something.

Group 2, participant U7

Watching television was emphasized in both groups, and activities related to cleaning and housekeeping were popular. Overall, participants in both groups seemed to like routines. The everyday life activity themes are detailed in Multimedia Appendix 6.

### The Needs of Older Adults for eHealth Services

#### Overview

The older adults’ needs for eHealth services were collected based on the main findings from remote, semistructured interviews and sentence completion tasks. The themes discovered from the interviews, the main findings under each theme, and the participants’ needs based on those findings are provided in Multimedia Appendix 7. In addition, sentence completion revealed the participants’ wishes and needs for eHealth services in the future (Multimedia Appendices 1 and 2). The needs described in the following sections were expressed based on the data.

#### The Need for a Carefree Mind

Older adults need a carefree mind when taking care of their health and regarding their current health and well-being situation, which can be supported with eHealth services. They need to understand the safety of eHealth service use and be able to manage health-related matters well regardless of the channel (eg, mobile phone, chat, or remote appointment). Users’ technical skills vary, and some use technical devices versatilely. Support and training possibilities help with this need. Feedback on one’s current health and well-being situation via eHealth services is appreciated. The safety needs are evident in the following quotes:

I avoid using all parts [of the user interface] that have even a little bit of a foreign language to me. Because it might happen there that I don’t understand something and make a wrong choice somehow [in the user interface].

Group 1, participant U2

I think managing my health-related affairs should be safe.

Group 1, participant U6

The need to obtain health care service regardless of the service channel is reflected, for example, in the following comments:

I would like to be able to use eHealth services in parallel with other service channels in the future.

Group 1, participant U1

I look for information on the internet every day. I use all possible medical and health services that are available digitally. And the same thing with public services: everything that is possible, I do them in digital channels.

Group 2, participant H3

On the basis of the data, people’s diversity is emphasized: being able to use the eHealth service can be a big challenge for one person, but for another, it brings benefits and ease. Owing to older adults’ varying technology skills and device availability, health self-management was desired in varying ways and different channels, either face-to-face or digitally. In addition, group 1 participants seemed to be experienced or very experienced with technology and felt very comfortable using eHealth services. The clear variations between the participant groups should be considered in all design decisions regarding eHealth service development.

Varying skills and comfort levels with using eHealth services are demonstrated, for example, in the following quotes:

For me, using eHealth services is natural and easy.

Group 1, participant U6

For me, using eHealth services is easy and self-evident.

Group 2, participant HX4

eHealth services do not help me because I have neither the equipment nor the skills.

Group 2, participant S8

Learning something new [about digital services] is difficult because of the tricky terminology. I’ve never studied English at school, and systems use a special system language. ...Then these jungles of safety encryptions and passwords: remembering them is almost the most difficult part of it.

Group 2, participant S9

#### The Need for eHealth Service Adaptation Based on Current Life Situation, Use Contexts, and Everyday Life

Regarding different life situations, the participants needed support via eHealth with their health and well-being in changing situations, such as when retiring, regardless of their current technology skills or devices. eHealth could help them, for example, with mental well-being or keeping physical activity levels high enough. The changing situations are evident in the following quotes:

My working years are in the final phase. It’s sure that I have a couple of years left until I retire officially. After that, my children are already adults as well. But currently, my life is quite work-oriented, long workdays, and I’m all the time busy.

Group 1, participant U1

I have done more or less work [in my life]. And now I’m retiring. That’s a pretty big change.

Group 1, participant U4

I’d like to use eHealth services in the future even after I retire.

Group 1, participant U12

I have a little bit tricky stage of life. My son went to heaven two weeks ago, and I am currently going through grief. Five years ago, my husband died. So, that kind of stage of life I have now.

Group 2, participant H2

Yes, it was easier when I was healthy. After all, then I could do what I wanted and run if I liked and, in every way. ...That life was completely different then. I can’t say, however, that none of these moments in this life have been completely unpleasant. ...When I think about this end of life, yes, I have had a good life the whole time, no matter what era it was. They have all been part of life that was then, and it was good at that moment.

Group 2, participant S7

Older adults desired eHealth services that could adapt to their current and evolving life situations, such as retirement or changes in health. These services should be tailored to their lives in terms of both content and functionality, including the user interface, and should be flexible enough to adjust according to the season, regardless of their location. Older adults want to take care of their health and well-being regardless of location (from places other than home or a clinic, such as a summer cottage). Service adaptation to everyday life makes eHealth services relevant.

However, the ease of use of an eHealth service becomes relevant only after it has been ensured that it can be accessed at all. One’s health state affects everyday life and the ability and willingness to use eHealth services. For example, when a health situation improves due to following healthy daily routines, the content of the eHealth service should follow this new health situation and its user’s new needs for the eHealth service. eHealth services should be adaptable to changes in life and health situations as well as fluctuations in motivation to use eHealth, both in the short and long term. eHealth service’s role and value as a part of changing life situations and user needs regarding eHealth service’s adaptability are reflected in the following quotes:

eHealth services help me very well in my health situation.

Group 1, participant U12

eHealth services help me plan schedules flexibly.

Group 1, participant U7

For me, the most important thing with eHealth services is that I don’t have to commit to a specific time.

Group 1, participant U10

For me, the most important thing in eHealth services is the right information at the right time.

Group 2, participant S9

For me, the most important thing with eHealth services is that I get information immediately without waiting.

Group 2, participant HX4

Using them [eHealth services] saves time.

Group 1, participant U8

#### The Need for a Holistic Perspective

Older adults need content that supports their health and well-being holistically. eHealth services should, for example, include perspectives on their current everyday lives and health situations. Group 1 members often wished that eHealth services would be one service among others, smoothly integrated into everyday life. For group 2 members, more research is needed to investigate their perspectives on a suitable package of service channels and content so that they receive the same holistic support as group 1 members. The example quotes behind the need for holistically offered health and well-being support as a part of older adults’ everyday lives are as follows:

I fill in crosswords, and I read when I like to. I cook, but not every day now. Things like that I get pleasure from. After all, all in all, the fact that there are not so many financial worries is a part of that balance. Everything you need is here and now. Everyone can ask themselves the question whether they are happy.

Group 1, participant U11

For me, the most important thing in eHealth services are the right health goals considering the right information; based on researched and measured information.

Group 1, participant U7

eHealth services help me to plan flexible schedules.

Group 1, participant U7

eHealth services help me take care of things related to my health at once. They save time. I get e.g., lab results through a digital service.

Group 1, participant U13

eHealth services help me get an overall picture of my health.

Group 1, participant U1

I read a lot. Now that I’ve been here sick and the moments, I’ve been awake, I’m reading something all the time.

Group 2, participant HX6

And then jogging with the dog. It keeps me in balance. All my social interaction now takes place there, walking the dog, because there are many dogs here, and we have lived here 45 years, so there are so many people who know dogs.

Group 2, participant S8

#### The Need to be Able to Avoid Using eHealth Services But to Use Specific eHealth Functions

Older adults, especially group 2 members, need to be able to select the service channel: eHealth services or face-to-face. On the other hand, there was a need for eHealth functions. For example, group 2 members often appreciated the opportunity to look at laboratory results without having to go anywhere. The COVID-19 pandemic era might have affected this result. The following example quotes bring insights into the older adults’ needs for specific functions and the possibility of obtaining health care services via service channels other than eHealth services:

In principle, they [service providers] accept to do business [face-to-face], but if you ask for some advice there, they recommend that you “go to do it online or look it up online.” They don’t understand that not everything works perfectly on the internet for them either. I think it’s pointless for them to guide you to do business online when it can’t be done there online.

Group 1, participant U8

Or you can use a video connection to contact the nurse! They are certainly good additions to the healthcare service.

Group 1, participant U1

It’s embarrassing that I don’t know how to book appointments [digitally]. I don’t know how to book a time for our laboratory or X-ray. I don’t know how to make an appointment. And you should know that, but when there was no need, you didn’t learn.

Group 2, participant HX6

When I imagine myself retired, and when I no longer have a work computer, and if it is difficult to get from one place to another, then of course, they [eHealth services] will be useful. And then the chat service, where you can easily ask for help or advice!

I think managing my health-related affairs should be possible in person.

Group 2, participant S8

This means that eHealth services should be accessible: (1) devices should be available and (2) the network connection should be stable enough, and the service must be available regardless of time. The service should be offered in a multichannel manner, including personal contact, phone service, or in writing (eg, chat).

Operations related to health and well-being, such as appointment booking through eHealth services, must be clear and easy to use for all user groups. The operations and the eHealth service should support long-term and short-term health and well-being planning in terms of the content and functions available to both end users and health care professionals. It should work reliably in all situations and support older adults’ empowerment in managing and controlling their current and future health situations. This is evident, for example, in the following quotes:

I think operations related to my health and well-being should be easy.

Group 2, participant HX5

eHealth services do not help me at all.

Group 2, participant HX6

#### User Needs Regarding User Experiences

The completed sentences revealed that the older adults’ experiences with eHealth services varied, with some finding them easy to use and others finding them challenging. The use of eHealth services was felt to be part of everyday life and routines, and it did not produce many “peak experiences,” which are the most memorable experiences producing positive emotions [70,80]. The variation in user experiences from eHealth use is apparent in the following example quotes:

For me, using eHealth services is easy, and a routine.

Group 1, participant U13

Using them [eHealth services] makes me a little irritated.

Group 1, participant U1

I think managing my health-related affairs should be confidential, and the response quick and empathetic.

Group 1, participant U11

Using them [eHealth services] puts me in a bad mood.

Group 2, participant HX6

Two (25%) out of 8 participants in group 2 had worrying experiences, such as a loss of self-confidence in the use of eHealth services and an increase in concern about the use of eHealth services. These worries are reflected in the following example quotes:

Using them [eHealth services] makes me feel insecure.

Group 2, participant S7

The thing that worries or scares me the most about eHealth services is that soon you won’t be able to see a doctor anymore when everything will be done digitally.

Group 2, participant S8

However, eHealth services especially brought peace of mind to group 1 participants, seeming to improve their well-being. For both groups, eHealth services produced important information about their current health situation. eHealth services seemed to be used in both age groups, not only out of necessity but also to obtain information and improve the flexibility of taking care of their health. eHealth services provided longer-term insights into their own health status, and information obtained through eHealth services was highlighted at different points. Finding out about their own health status seemed important. With the help of eHealth services, the level of concern about their own health condition improved. Group 2 members had more negative views of eHealth services, and they also mentioned a lack of equipment as a barrier to eHealth service use. eHealth service’s role in offering information, flexibility in taking care of one’s health, or role as a calming factor is reflected in the following example quotes:

I use eHealth services if I book an appointment with my doctor, or check my health information

Group 1, participant U12

I use eHealth services whenever possible.

Group 1, participant U6

Using them gives me peace of mind when I can book appointments and find out about health-related issues at a time that suits me.

Group 1, participant U13

Other barriers to eHealth services were lack of human contact, inefficiency, and challenges with its use. eHealth services divided participant opinions regarding its benefits: group 1 participants did not have as many helpful experiences with eHealth services as group 2 participants. Both groups included individuals who felt that eHealth services did not provide holistic support during life changes. At the same time, group 2 participants felt that eHealth helped them acquire information about health-related issues, supported them in planning health-related issues, and helped them commit to lifestyle changes. On the other hand, they lacked competence and felt that eHealth services (use processes and user interfaces) was too complicated. The negative experiences and use barriers are evident in the following quotes:

With eHealth services, I am saddened by the lack of consistency and aimlessness, as well as the lack of genuine and cheerful support.

Group 1, participant U7

With eHealth services, I am frustrated by delays and waiting.

Group 1, participant U6

With eHealth services, I am saddened by my laziness and lack of time available to study the use of eHealth services.

Group 2, participant H3

With eHealth services, it saddens me that I don’t know how to use them.

Group 2, participant HX6

The most important things in eHealth services use were efficiency, ease and smoothness of transactions, access to information, planning (where group 1 members had diverse answers), and support for well-being. Participants in group 2 had the same eHealth needs as those in group 1 but they were more skeptical (Multimedia Appendix 8).

The efficiency, ease, and support for well-being via eHealth services are apparent in the following quotes:

Using them [eHealth services] saves my time.

Group 1, participant U6

Using them [eHealth services] makes me think about my health based on researched information.

Group 1, participant U10

For me, the most important thing about eHealth services is that I don’t have to commit to a specific time or date.

Group 1, participant U10

eHealth services help me renewing my medicine prescriptions.

Group 2, participant S7

## Discussion

This qualitative exploratory study aimed to gain a more in-depth understanding of older adults’ needs for eHealth services as part of their everyday lives in different life stages. The data were collected with interviews and a cultural probe package, including 4 background information questionnaires, sentence completion tasks, and a diary-based exercise.

### Principal Findings

#### Everyday Life of Older Adults

Both participant groups valued time spent at home and home-based activities as part of their everyday lives. Healthy eating habits were an especially important aspect of the participants’ well-being from their own perspective. These older adults enjoyed different activities, such as jogging and Nordic walking, which is in line with other literature [31].

Older adults generally appreciated bringing health and well-being into everyday life. They had weekly or daily activities or hobbies that supported them to reach their long-term health targets. These routines varied greatly, including exercise, good night sleep, personal hygiene, repetitive food, pet care, and housework-related morning routines. Group 1 participants reported routines and activities that partly differed from those of group 2 participants. For example, all the routines related to the gym or exercising while commuting were reported by group 1 participants. Interestingly, the only mention of reading newspapers with a tablet was reported by a participant from group 2. Furthermore, a mention of doing morning exercise with television was reported by a group 2 participant. Cleaning routines were mainly mentioned by participants from group 2.

The eHealth service can both support health self-management on a long-term basis and activate and support older adults in ad hoc type of activities and operations related to health and well-being, for example, by offering information based on the current health situation or reminding them of a (daily or weekly) hobby.

#### The Needs of Older Adults for eHealth Services

On the basis of the interviews and sentence completion tasks, four needs for eHealth services were identified: (1) the need for a carefree mind; (2) the need for eHealth service adaptation based on the current life situation, use contexts, and everyday life; (3) the need for a holistic perspective; and (4) the need to avoid using eHealth services but to use specific eHealth service functions. These needs should be considered when designing eHealth services that support older adults’ healthy living. In addition, they generally apply to both user groups but may be emphasized differently depending on the group.

On the basis of our findings, the older adults seemed to have generally positive attitudes toward eHealth, aligning with those identified by Cabrita et al [15] and Mielonen et al [28]. The infrastructure behind eHealth services must be stable; then, eHealth services can be offered in an accessible way and without limitations for everyone, assuming that end devices are available. Owing to varying end devices among older adults, eHealth services should be scalable regardless of the service channel or devices. Therefore, the design should support the reliability of eHealth services so that they work in all situations, supporting older adults’ health self-management [15,70]. Our study adds the perspective of changing time spans: the service must function smoothly for both short-term and long-term use, accommodating variations in use duration.

Well-being, sickness, health, and illness seem to be subjective personal experiences. The need for eHealth services changes due to the user’s current health and well-being situation: eHealth services become unnecessary when health and well-being increase. Therefore, it must be possible for older adults to easily try out whether eHealth services will benefit them. An eHealth service may also be used less frequently at times.

eHealth services can support older adults’ health and well-being, particularly when the content of the service aligns with their everyday lives and interests. Therefore, we recommend that the eHealth service work as part of an older adult’s healthy routines, which might include, for example, encouraging attitudes and functions for housework and other activities.

### Comparison With Prior Work

Our results show that understanding the user’s life stage and the key interests at that stage is important, as found in other literature [47]. Owing to older adults’ changing health and life situations [1-3,10,11,47], an individual eHealth service should function as part of a wider service offering so that the service supports and adapts to changing life situations and health statuses. For example, in this study, it was recognized that both those who are still working and those who are retired use IT and eHealth services. IT use at work might support learning new skills and sometimes even updating them [70]. In addition, eHealth service flexibility is valued [4]. The need for eHealth services to adapt to the older adult’s current life and health situation is in line with the study by Reiners et al [81].

Earlier research recognized a large variation in activities that support older adults’ well-being, in line with our findings: exercising; cooking; and hobbies, such as language courses [82]. Even with poor health, older adults achieve well-being and positive effects from daily activities [82,83]*.* In addition to physical activities, daily activities, such as reading books or watching television, can also offer relaxation [81]. Owing to the differing routines and daily activities, the need for eHealth adaptation based on the current life situation, use contexts, and everyday life was recognized in this study, and we recommend that eHealth services work as a part of older adults’ daily living smoothly. Previous research supports our results behind this user need: older adults are a heterogeneous group whose activity possibilities vary regarding their living conditions or current health situation [31]. However, the importance of studying the use of eHealth service as part of everyday life has been recognized [19].

The devices with which the eHealth services are used should be considered when designing these services. Given the possible effect of the small sample size, it can be seen from the data that group 2 participants seemed to use eHealth services somewhat less than group 1 participants. One reason for that could be that older adults who are still employed might use eHealth services for work reasons, as well [70]. On the other hand, it can probably be expected that, nowadays, older adults who are retired have used at least some IT at work.

Understanding the safety of eHealth services use was important to older adults, as in earlier research [4,5,10,18]. In line with previous literature [18,70], they needed available support and training for eHealth service use. In particular, the older participants in this study also needed not to be forced to use the eHealth service. However, this varied considerably between participants, which supports earlier research results [18].

In line with an earlier study [15], one of the most important design functions is to ensure that the eHealth service conveys empathy to older adults. This can be offered via an empathic tone of voice in the services and by offering them through service channels that are especially suitable to each older adult’s current situation. The whole life span, including transitions to different life phases, such as retirement, should be considered in design decisions [2,47,70]. This can be done, for example, by offering relevant content related to health and well-being to different older adult groups and by making sure that the content is relevant to each end user’s unique life and health situation.

On the basis of our findings, both those still working and those who have already retired have suitable devices and sufficient technical skills, as also reported by Mielonen et al [28]. However, eHealth services were used only by half (4/8, 50%) of the older participant group. Our findings support the observations of Kruse et al [3] regarding common barriers to eHealth use among older adults: a lack of self-confidence, a fear of making irreversible mistakes, and content that does not meet their needs. Fear can be dispelled with a clearer service concept, implementation support, user support, and instructions, as well as reassurance, such as by highlighting safety both in the service content and in the presentation of the service. In addition to minimizing fear, strengthening the self-confidence of the end user of the service plays an important role. Another phenomenon that emerged from the data was that well-being, health, and illness seem to be subjective, personal experiences.

The need for eHealth services changes due to the user’s current health and well-being situation; eHealth services become unnecessary when health and well-being improve. Therefore, it should be possible for older adults to easily try out whether the eHealth service will benefit them. This can be done, for example, by offering trial versions of services, which other research [15,18,84] recommends. Being able to safely try out an eHealth service could help reduce fears, build self-confidence, and better identify the current need for the service. Another helpful option would be to group service functions into easier basic functions and possibly other more complex functions that are recommended to be familiarized with time, as is recognized in the literature [15,47]. However, based on the study findings, the end user’s age or life situation does not appear to be a decisive factor in determining the need for user interface content. That said, older adults who are retired may face slightly more challenges in using eHealth services compared to those who are still working.

As also found in other literature [18,70], individual eHealth service should function as part of a wider, multichannel service package so that it adapts to changing life situations and changes in health status. It should work as a channel for end users to receive empathy in challenging health situations. This could be done with effective but sensitive communication possibilities and with empathic content. Because eHealth services should support older adults’ health and well-being, it is important to look at and connect their well-being in the IT context [84]. This can be done according to content and with easy and logically functioning user interfaces. In addition, this study’s participants wished for an option to not use eHealth services or specific eHealth functions. This need is in line with other research findings [85].

### Limitations

This qualitative exploratory study aimed to gain a more in-depth understanding of older adults’ needs. The sample was small; the participants were from 1 country, and they were likely to be more interested than average in eHealth services. Older adults are a diverse group from the perspective of, for example, cognitive, motor, or technology use abilities [61]. Therefore, completely covering the population with a representative sample was not possible. This has potentially biased the results. In addition, the COVID-19 pandemic era could have influenced the results, but this was not especially followed as a part of this study.

Regarding the adaptation of eHealth services to different life and health situations, we do not know whether the service should always be the same or differ depending, for example, on whether the health situation has changed, in which case the role and meaning of the service changes from supporting well-being to treating illness. We recommend this as a future research area, especially in long-term studies. More research is needed on the best service channel combinations for older adults to obtain sufficient holistic health and well-being support. In the future, it would be worthwhile to test the design recommendations with representatives of the designer community and older adults, for example, in co-design workshops. In this study, the older adults’ needs in different life situations were not compared to those of other age groups. However, this would be valuable to study in the future.

From a methodological perspective, the cultural probe package, including diary-based and sentence completion tasks, combined with remotely conducted semistructured interviews seemed to be effective in collecting information about participants’ everyday lives and in mapping user needs for eHealth services. This is interesting because, as far as we know, not many cultural probes studies on this topic are available. The working participants completed more handwriting-based sentences. Therefore, cultural probes including many handwriting exercises might fit better for them. On the other hand, the data were collected using several methods, which confirmed the findings of the study and helped identify user needs. The methods seemed to work well in remote conditions during the COVID-19 pandemic era.

### Conclusions

In this qualitative study, we collected information about the everyday lives of older adults in different life situations and identified their related needs for eHealth services. The results helped us understand how older adults see and experience health care with the help of eHealth services as part of everyday life. The older adults in this study often preferred a consistent routine in their daily practices including healthy eating and exercise. eHealth services could offer time saving and flexibility within these routines. A lack of devices or skills was often mentioned as a use barrier. On the basis of the results, four main needs of the older adults for eHealth services were identified: (1) the need for a carefree mind, in which older adults could receive status information on their current health and well-being situation via eHealth services; (2) the need for eHealth service adaptation based on the current life situation, use contexts, and everyday life; (3) the need for a holistic perspective, in which older adults wished to receive support for health issues from many perspectives and through several different service channels; and (4) the need to avoid using eHealth services but to use specific eHealth service functions, in which face-to-face support for their health was especially appreciated. On the basis of the results, eHealth services should be designed such that they fit well in older adults’ everyday lives and adapt to users’ everyday practices and health statuses according to content. eHealth services should be accessible and reliable.

## Appendix

Multimedia Appendix 1 Sentence completion examples.

Multimedia Appendix 2 Sentence completion task by age group (current situation).

Multimedia Appendix 3 Remote, semistructured interview (translated from Finnish).

Multimedia Appendix 4 Interview data analysis code comparison and final codes after comparison.

Multimedia Appendix 5 Health Confidence Score, eHealth Literacy Scale, and European Health Literacy Survey Questionnaire results by age group.

Multimedia Appendix 6 Identified themes in life activities.

Multimedia Appendix 7 Interview themes, example quotes, and user needs.

Multimedia Appendix 8 Differences in the completed sentences between the 2 groups, focusing on the situation in the future.

## Abbreviations

DigiIN: Toward a Socially Inclusive Digital Society: Transforming Service Culture Project
eHEALS: eHealth Literacy Scale
HCS: Health Confidence Score
HLS-EU-Q16: European Health Literacy Survey Questionnaire

## Data Availability

The datasets generated and analyzed during this study are not publicly available due to the sensitive nature of the data, but the numeric data are available from the corresponding author on reasonable request.
