# Supplementary material for: Exploring Older Adults’ Needs for a Healthy Life and eHealth: Qualitative Interview Study
Source: JMIR Hum Factors. 2025 Jan 8;12:e50329. doi: 10.2196/50329 (PMC11754987; doi:10.2196/50329)
Supplement: Multimedia Appendix 1 [file humanfactors_v12i1e50329_app1.pdf]

Incomplete sentences	Response examples [a]
**eHealth services now**	
	For me, using eHealth services is…<br><br>1A: … easy and routine.<br>1B: …less exciting, but of course it's worth learning how to use them—they are the future.<br>2A: …easy and obvious.<br>2B: …very challenging.
	Using them makes me…<br><br>1A: …think about my health based on the information available.<br>1B: …feel better.<br>2A: …interested in electronic services.<br>2B: Using them puts me in a bad mood.
	Related to eHealth services, I am bothered by…<br><br>1A: …the lack of consistency and aimlessness, as well as the lack of genuine and cheerful support.<br>1B: …when they are sometimes too complicated.<br>2A: …the fact that I don't know how to use them.<br>2B: … laziness and lack of time to study the available services.
	eHealth services help me…<br><br>1A: …to get an overall picture of the situation.<br>1B: …to save time and effort.<br>2A: …to get information about my diseases and medicines.<br>2B: …to get the information I need at any given time.
	eHealth services do not help me…<br><br>1A: …if I want to find out something detailed.<br>1B: …to ask for advice.<br>2A: …get younger.<br>2B: …because I have neither the equipment nor the skills.
	I use eHealth services if…<br><br>1A: …whenever possible.<br>1B: …I need a doctor or nurse and want to check my situation.<br>2A: …I need or find it interesting to know.<br>2B: I would use eHealth services if someone taught me about them.
	For me, the most important thing in eHealth services is…<br><br>1A: …ease of use and reliability.<br>1B: …that I can take care of things from home or work.<br>2A: …that I get information immediately without waiting.<br>2B: …the right information at the right time.
**eHealth services in the future**	
	I would like to be able to use eHealth services in the future…<br><br>1A: …in parallel with other service channels.

	1B: I would like to be able to use eHealth services more and more versatilely in the future.<br>2A: …at least on the same scale as now.<br>2B: I would like to be able to use eHealth services to find information and service offerings in the future.
	I think dealing with my health should be…<br><br>1A: …confidential, the response quick and empathetic.<br>1B: …done both electronically and in person.<br>2A: …a clear and consistent progression online.<br>2B: ...possible in person with people.
	If eHealth services are available by phone, I…<br><br>1A: …would definitely use them.<br>1B: …will be using them more and more.<br>2A: …would use the services every day.<br>2B: …want a person to answer, not a machine.
**Concerns and fears for eHealth**[b]	
	I am worried or afraid to use eHealth services because…<br><br>2A: …I do not know how to do it, and I do not have the equipment to use it.<br>2B: …I do not know how to use it.
	What worries me or scares me the most in eHealth services is…<br><br>2A: …incompetence!<br>2B: …that you won't be able to see a doctor anymore; everything will be electronic.
	I don't use eHealth services because...<br><br>2A: …I don't know how to do it!<br>2B: …I don't know how to do it, and I don't trust it either.

[a] Younger participant group response examples 1A and 1B/ Older participant group response examples 2A and 2B.

[b] Asked only of older participant group participants
