# Supplementary material for: Exploring Older Adults’ Needs for a Healthy Life and eHealth: Qualitative Interview Study
Source: JMIR Hum Factors. 2025 Jan 8;12:e50329. doi: 10.2196/50329 (PMC11754987; doi:10.2196/50329)
Supplement: Multimedia Appendix 2 [file humanfactors_v12i1e50329_app2.pdf]

Number and contents of the completed sentences			
	Group 1 (n = 12)	Group 2 (n = 8)	Comments
For me, using eHealth services is…	12	5	The participants were used to using eHealth, but their feelings varied.
Using them makes me…	11	5	The older group's answers were more negative.
Related to eHealth services, I am bothered by…	11	5	The same things upset both groups, but the younger ones would have liked more consistency. The only positive response was among the older participant group. The respondent was satisfied with electronic health services and almost never bothered by them.
eHealth services help me…	11	4	The younger participant group felt that eHealth services were engaging and brought smoothness and planning possibilities, while only the older participant group felt that acts for better health and well-being were easier.
eHealth services do not help me…	6	3	In the case of the younger participant group, the uselessness was because the health goal cannot be solved with the help of an electronic service, but requires real measures, while the older participant group experienced poor usability, complexity, and a lack of equipment.
I use eHealth services if…	11	4	The same content in both participant groups concerned the need for eHealth services as such and a thirst for knowledge. The younger participant group's answers were significantly more positive, while the older participant group also perceived complexity, difficulty, poor usability, and lack of equipment in this category.
For me, the most important thing in eHealth services is…	11	5	The older participant group had a thirst for knowledge and the experience of not being able to benefit from eHealth services, while the younger participant group saw many benefits of eHealth services. Both groups felt that acts for better health and well-being became easier and more efficient. Reliability was important to both groups.
Total	73	31	
Proportion of sentences in all	70%	30%	
