# Supplementary material for: Exploring Older Adults’ Needs for a Healthy Life and eHealth: Qualitative Interview Study
Source: JMIR Hum Factors. 2025 Jan 8;12:e50329. doi: 10.2196/50329 (PMC11754987; doi:10.2196/50329)
Supplement: Multimedia Appendix 3 [file humanfactors_v12i1e50329_app3.pdf]

Warm-up questions

I am interested in your computer use habits at the beginning of this interview.

- What kinds of things do you use the computer for (at work and in your free time)?
- What kinds of things are easy for you on the computer?
- What kinds of things seem difficult?
- What things do you like to do on the computer? What things, on the other hand, perhaps even seem suspicious or scary on a computer?
- What causes fear?
- (If you don't use a computer): What difficulties have you encountered because you don't use a computer?

Theme: Me and my everyday life

As background information for this interview, I would like to ask about your stage of life. What phase of life are you going through?

- Optional: How do you feel about your health and well-being now? / How satisfied are you with your health and well-being?
- Optional: (What are you satisfied with regarding your health and well-being?)
- Optional: (What problems do you experience in taking care of your health and well-being?)
- Optional: (What about practical matters in health care?)
- (Additional question: Do you know when you are going to retire? What do you think about retirement in terms of health and well-being?)

Now, let's move on to the topics in the exercise book (cultural probes) and continue discussing them.

- What is your typical day like?
- Which people do you meet in your everyday life, and under what circumstances?
- What everyday things, choices, and routines do you do to support your health and wellbeing?
- What kinds of things, choices, and routines do you do in your everyday life to support your mental well-being?

Theme: Long-term well-being

- There was a task in the exercise book (cultural probes) where you could draw a timeline related to well-being. How has your health situation changed over time?
- (Optional: Was there any factor that improved or worsened your well-being?)

Theme: Usefulness of eHealth services

- In your opinion, what benefits could eHealth services have for you now?
- What benefits do you think eHealth services could have in the future?
- What kinds of eHealth services do you think are not useful now and in the future?
