# Supplementary material for: Exploring Older Adults’ Needs for a Healthy Life and eHealth: Qualitative Interview Study
Source: JMIR Hum Factors. 2025 Jan 8;12:e50329. doi: 10.2196/50329 (PMC11754987; doi:10.2196/50329)
Supplement: Multimedia Appendix 4 [file humanfactors_v12i1e50329_app4.pdf]

Codes of the researcher 1	Codes of the researcher 2	Comparison; the final codes
Life and health situation	Everyday life activities	Life and health situation
Possible health challenge	Retiring	Possible health challenge
Devices in use	Life situation	Technology use
For what to use devices	Easy with the technology	Easy with the technology
Applications in use	Bad with the technology	Challenges with technology
Easy to use	Increasing unwell	For what to use devices
Challenges in use	Good with the technology	Technology skills
Fear and unpleasant with technology	Family care	Fear and unpleasant with technology
Nice and pleasant with technology	Tested eHealth service	Tested eHealth service
The moment of retirement	Fear with the technology	The moment of retirement
Health and well-being related customer experiences	Positive	Healthcare
Customer experiences (face-to-face, remotely)	Stakeholders	Health services
Acts for health and well-being	Use of technology	Acts for health and well-being
Acts for mental health	Technology skills	Family care
Social contacts	Healthcare	Support from technology
End-user needs	Bad health	Stakeholders
Health services in the future	Good health	Health services in the future
	Health services	Health and well-being related customer experiences
	In the future	Applications in use
	Work life	Nice and pleasant with technology
	Challenges with technology	End-user needs
		Customer experiences (face-to-face, remotely)
