# Supplementary material for: Exploring Older Adults’ Needs for a Healthy Life and eHealth: Qualitative Interview Study
Source: JMIR Hum Factors. 2025 Jan 8;12:e50329. doi: 10.2196/50329 (PMC11754987; doi:10.2196/50329)
Supplement: Multimedia Appendix 5 [file humanfactors_v12i1e50329_app5.pdf]

	Group 1, Mean (SD)	Group 2, Mean (SD)	All participants, Mean (SD)
eHEALS, eHealth Literacy Scale (scale 1–5)	3.6 (1.3)	2.6 (1.7)	3.3 (1.5)
the European Health Literacy Survey Questionnaire, HLS-EU-Q16 (scale 1–4)	2.9 (1.4)	3.0 (0.9)	2.9 (1.2)
Health Confidence Score (HCS) (scale 0–3)	2.4 (0.6)	2.4 (0.56)	2.4 (0.6)
