# Supplementary material for: Exploring Older Adults’ Needs for a Healthy Life and eHealth: Qualitative Interview Study
Source: JMIR Hum Factors. 2025 Jan 8;12:e50329. doi: 10.2196/50329 (PMC11754987; doi:10.2196/50329)
Supplement: Multimedia Appendix 6 [file humanfactors_v12i1e50329_app6.pdf]

Theme	Number of mentions	Theme contents
Total	622	
Spending time at home	102	Most commonly, participants watched TV and read at different times of day. This theme also included listening to audiobooks and radio. Spending time at home was also emphasized in general.
Dining and cooking	96	Food included different routines among the participants. Breakfast and lunch were usually mentioned as routines, with more variation related to food towards the evening. The importance of food for well-being and the participants' attitudes toward food (routine, medical care, pleasure, hobby, etc.) were often mentioned.
Routines	74	Routines included bedtime activities and activities intended to guarantee a good night's sleep, morning routines, personal hygiene, cleaning, and housework.
Hobbies or exercising	72	Exercising was popular. Walking and jogging were commonly practiced, including Nordic walking and climbing stairs. Walking could also be a social activity with a partner or a dedicated group. Water exercise and swimming, going to the gym, body care, and golf were mentioned by several participants. Volunteer work and intellectual activities were also practiced. Opportunities for exercise were actively and creatively sought. Everyone seemed to have ways of exercising that they liked.
Sleep	51	The participants were interested in sleep quality. Some reported waking up in the middle of the night, waking up in general, bedtime routines, and the length of the night.
Mundane activities	51	The most often mentioned mundane activity was going to the store, which was sometimes combined with other activities, such as going to the post office or having lunch or coffee. Walking was often mentioned as a part of these activities.
Work-related	43	Some participants reported work as routine or on a very general level. However, others reported work being hectic, accomplishing a lot during the workday, or learning new things.
Medical treatment	23	Medical treatment related activities included taking medication, spreading lotion on one's feet (as a part of treatment), measuring blood sugar, or visiting the doctor.
Cottage or nature	23	Activities conducted at summer cottages included warming the cottage with firewood (including cutting and carrying the firewood) or warming the sauna. This theme included activities clearly related to nature, such as walking in the forest and picking mushrooms.

Friends	20	Contact with friends happened through meeting them, phone calls, and messages. One participant had organized a virtual coffee meeting with their friends.
Pets	17	Activities related to taking care of their dog, including mostly walking but also feeding the dog.
Pampering and rest	17	Activities were intended to increase the participants' well-being but were also necessary, such as resting and napping. For some participants, this was a necessity, and for others, it was more relaxing.
Communication with relatives	13	Keeping in contact with children and grandchildren.
Planning and controlling everyday life	10	Activities related to preparing lunch (to take to work), packing and preparing for a short trip, and planning as well as preparing daily meals.
Weather	10	Several participants mentioned the weather as something that affected their daily well-being or activities.
