# Supplementary material for: Exploring Older Adults’ Needs for a Healthy Life and eHealth: Qualitative Interview Study
Source: JMIR Hum Factors. 2025 Jan 8;12:e50329. doi: 10.2196/50329 (PMC11754987; doi:10.2196/50329)
Supplement: Multimedia Appendix 7 [file humanfactors_v12i1e50329_app7.pdf]

Theme	Example quotes from the Group 1 (n = 180).	Example quotes from the Group 2 (n = 115).	User needs
**Life situation**			
Life situation	My working years are in the final phase. It's sure that I have a couple of years left until I retire officially. After that, my children are already adults as well. But currently, my life is quite work-oriented, long workdays, and I'm busy all the time. (Participant U1)	I have a little bit tricky stage of life. My son went to heaven two weeks ago, and I am currently going through grief. Five years ago, my husband died. So, that kind of stage of life I have now. (Participant H2)	• Need for eHealth adaptation based on the current life situation, use contexts, and everyday life.<br>• Need for a holistic perspective.
Use of devices and technology	Well, I use it [the computer] day and night! I'm working in such a position that I need to do that. And I have used it [the computer] already for years. Well… then there are [in the study's questionnaires] such questions like "Do you use the internet?" I use the internet all day long! I search for information, I order different kinds of things, and I arrange things, anything! (Participant U13)	I prefer doing business with a computer rather than a mobile phone. The content is bigger on the computer than on the mobile phone screen. My fingers are clumsy, so error keystrokes will be made on the mobile phone. (Participant H3)	• The Need to Avoid Using eHealth Services But to Use Specific eHealth Functions
Technology skills	I use [computer, mobile phone] a lot. So, I don't have my eyes on the screen all the time, but I use it specifically to search and find information. (Participant U7)	I'm the administrator of a few associations' websites. I made them once decades ago, and I haven't found a maintainer for them, so I still must keep them around. (Participant S9)	• Need for a carefree mind.
Increasing mental well-being habits	I fill in crosswords, and I read when I like to. I cook, but not every day now. Things like that I get pleasure from. After all, all in all, the fact that there are not so many financial worries is part of that balance. Everything you need is here and now. Everyone can ask themselves the question whether they are happy. (Participant U11)	I read a lot. Now that I've been here sick and the moments I've been awake, I'm reading something all the time. (Participant HX6)	• Need for a carefree mind.<br>• Need for a holistic perspective.

Increasing physical well-being habits	I usually start work at 9.00, then breakfast, and then walking the dogs. I sit at the computer from 18.00–20.00. Somewhere in between food and coffee. At 16:00, feeding the dogs. At the end of the day, I don't meet anyone except dog walkers. I also meet new employees in my everyday life. (Participant U6)	And then jogging with the dog. It keeps me in balance. All my social interaction now takes place there, walking the dog, because there are many dogs here, and we have lived here 45 years, so there are so many people who know dogs. (Participant S8)	• Need for a holistic perspective.
Stakeholders	Who is in my core group? Well, I have one daughter, one son, a boyfriend, and a sister. And my sister and I don't live in the same place, so we don't see each other very often; we just call or text each other. (Participant U2)	I met my contacts from the associations. They have become good friends to me over the decades. But the COVID-19 period has reduced regular meetings, but I don't think it has brought any loneliness to me. I enjoyed myself very well, too. In my former hometown, no friends were nearby, so I've learned to enjoy myself alone. (Participant S9)	• Need for a holistic perspective.
Thoughts of retiring and healthcare services	I have done more or less work [in my life]. And now I'm retiring. That's a pretty big change. (Participant U4)	Yes, it was easier when I was healthy. After all, then I could do what I wanted and run if I liked and, in every way. […] That life was completely different then. I can't say, however, that none of these moments in this life have been completely unpleasant. […]. When I think about this end of life, yes, I have had a good life the whole time, no matter what era it was. They have all been part of life that was then, and it was good at that moment. (Participant S7)	• Need for eHealth adaptation based on the current life situation, use contexts, and everyday life.<br>• Need for a holistic perspective.
**Attitudes toward technology**			
Challenges with technology	In principle, they [the service providers] accept to do business [face-to-face], but if you ask for some advice there, they recommend that you "go to do it online or look it up online." They don't	Learning something new [about digital services] is difficult because of the tricky terminology. I've never studied English at school, and systems use a special system language. […] Then these jungles of safety	• Need for a carefree mind.<br>• The Need to Avoid Using eHealth Services But to Use Specific eHealth Functions.

	understand that not everything works perfectly on the internet for them either. I think it's pointless for them to guide you to do business online when it can't be done there online. (Participant U8)	encryptions and passwords: remembering them is almost the most difficult part of it. (Participant S9)	
Easiness aspect of technology	Official services and bank services and such are easy! When using them, you notice that product development has been done. They are quite user-friendly, and they guide the user to make the right choices to operate correctly. (Participant U10)	I look for information on the internet every day. I use all possible medical and health services that are available digitally. And the same thing with public services: everything that is possible, I do them in digital channels. (Participant H3)	• Need for a carefree mind.<br>• The Need to Avoid Using eHealth Services But to Use Specific eHealth Functions.
Downsides (including fear) of technology	I avoid using all parts [of the user interface] that have even a little bit of a foreign language to me. Because it might happen there that I don't understand something and make a wrong choice somehow [in the user interface].<br>(Participant U2)	It's embarrassing that I don't know how to book appointments [digitally]. I don't know how to book a time for our laboratory or X-ray. I don't know how to make an appointment. And you should know that, but when there was no need, you didn't learn. (Participant HX6)	• Need for a carefree mind.<br>• The Need to Avoid Using eHealth Services But to Use Specific eHealth Functions.
**Healthcare and eHealth**			
Healthcare services in the future	When I imagine myself retired, and when I no longer have a work computer, and if it is difficult to get from one place to another, then of course, they [eHealth services] will be useful. And then the chat service, where you can easily ask for help or advice! Or you can use a video connection to contact the nurse! They are certainly good additions to the healthcare service. (Participant U1)	Now that all services are offered remotely, these remote services should be quite simple, and unfortunately, they are not always. (Participant HX5)	• Need for eHealth adaptation based on the current life situation, use contexts and everyday life.<br>• Need for a carefree mind.<br>• Need for a holistic perspective.<br>• The Need to Avoid Using eHealth Services But to Use Specific eHealth Functions.
Healthcare and the moment of retiring	The easiest thing would be for all [health services] to continue in the same way even after retirement. It would be the same place and the same services. It	No data available	• Need for eHealth adaptation based on the current life situation, use contexts, and everyday life.

	seems that it would be more convenient if you could continue in the place of a private service provider instead of public health services. (Participant U4)		• Need for a carefree mind.<br>• Need for a holistic perspective.<br>• The Need to Avoid Using eHealth Services But to Use Specific eHealth Functions.
**Customer experiences**			
Experiences with healthcare (digitally, face-to-face)	A pike bit my hand, and the wound [got] infected, and my hand swelled badly. I was at the summer cottage on Saturday, and I got an antibiotic prescription via chat. I didn't have to go and wait in line for hours at the emergency room. […] all eHealth is a positive thing! (Participant U4)	They [the service providers] want all kinds of preliminary information to be filled in, at least 10 pages. They also claim that all information is in one place. Yes, I understand that background information is needed, but I filled out background information a while ago at a nearby hospital. […] I was thinking, where did they put the preliminary information in the nearby hospital when I must fill out the same information again? (Participant HX6)	• Need for a holistic perspective.<br>• The Need to Avoid Using eHealth Services But to Use Specific eHealth Functions.
