# Supplementary material for: Exploring Older Adults’ Needs for a Healthy Life and eHealth: Qualitative Interview Study
Source: JMIR Hum Factors. 2025 Jan 8;12:e50329. doi: 10.2196/50329 (PMC11754987; doi:10.2196/50329)
Supplement: Multimedia Appendix 8 [file humanfactors_v12i1e50329_app8.pdf]

Number and of the completed sentences			
	Group 1	Group 2	Comments of the contents
I would like to be able to use eHealth services in the future…	12	6	The younger participants were interested doing acts for better health and well-being remotely, while the older participant group brought up comprehensiveness and their need for knowledge, as well as the fact that they don't feel they will benefit of eHealth services in the future.
I think dealing with my health should be…	11	5	Both groups agreed on personal content in the eHealth service, ease, efficiency, possibility to plan activities for health and wellbeing, satisfying the need for information and good usability.
If eHealth services are available by phone, I…	11	6	Ease of use and using services more and more widely were the same between both groups.
Total	34	17	
Proportion of sentences in all	67 %	33 %	
